# The biodiversity Composition of Microbiome in Ovarian Carcinoma Patients

**DOI:** 10.1038/s41598-018-38031-2

**Published:** 2019-02-08

**Authors:** Bo Zhou, Chaoyang Sun, Jia Huang, Meng Xia, Ensong Guo, Na Li, Hao Lu, Wanying Shan, Yifan Wu, Yuan Li, Xiaoyan Xu, Danhui Weng, Li Meng, Junbo Hu, Qinglei Gao, Ding Ma, Gang Chen

**Affiliations:** Cancer Biology Research Center, Tongji Hospital, Tongji Medical College, Huazhong University of Science and Technology, Wuhan, Hubei 430030 China

## Abstract

Ovarian carcinoma is caused by multiple factors, but its etiology associated with microbes and infection is unknown. Using 16S rRNA high-throughput sequencing methods, the diversity and composition of the microbiota from ovarian cancer tissues (25 samples) and normal distal fallopian tube tissues (25 samples) were analyzed. High-throughput sequencing showed that the diversity and richness indexes were significantly decreased in ovarian cancer tissues compared to tissues from normal distal fallopian tubes. The ratio of the two phyla for *Proteobacteria*/*Firmicutes* was notably increased in ovarian cancer, which revealed that microbial composition change might be associated with the process of ovarian cancer development. In addition, transcriptome-sequencing (RNA-seq) analyses suggested that the transcriptional profiles were statistically different between ovarian carcinoma and normal distal fallopian tubes. Moreover, a set of genes including 84 different inflammation-associated or immune-associated genes, which had been named as the human antibacterial-response genes were also modulated expression. Therefore, we hypothesize that the microbial composition change, as a novel risk factor, may be involving the initiation and progression of ovarian cancer via influencing and regulating the local immune microenvironment of fallopian tubes except for regular pathways.

## Introduction

Ovarian cancer is the fifth leading cause of cancer death among women in western countries and is the most lethal gynecologic malignancy, especially high-grade serous ovarian cancer (HGSOC)^1^. Due to advanced stage diagnosis of the majority of patients, the 5-year-survival rate has not improved over the past few decades, remaining at just 45%, despite the standard first-line therapy of aggressive cytoreductive surgery followed by chemotherapy with paclitaxel, platinum-based agents, or a combination of these treatments^1^. Currently, serous ovarian cancer can be classified into two groups, Type I and Type II based on the clinical behavior, pathology, molecular genetics and tumor precursors^2,3^. Type I cancers are considered low-grade serous cancers, whereas Type II cancers are considered high-grade and constitute the majority of epithelial ovarian cancers, lack well-defined precursor lesions and are characterized by highly aggressive neoplasms^4,5^. However, the origins and molecular pathogenesis of high-grade serous ovarian cancers are still largely unknown^6,7^. Over the past decade, new evidence has challenged the theory that serous ovarian cancer originates from the ovarian surface epithelial. Instead of the ovary, the fallopian tube has been identified as a source of high-grade serous ovarian cancers^7–9^. The distal region of the fallopian tube, known as the fimbria, are exposed to the pelvic cavity, which is the most common location for serous carcinoma in BRCA-positive women with p53 mutations^10–12^. Owing to the exposure of the fimbria to pelvic cavity and extracorporeal linkage via uterovaginal passage, we hypothesized that carcinogenesis may be promoted or driven by several factors, including but not limited to pelvic inflammatory disease (PID), the tumor local immune microenvironment, hormonal fluctuations and spontaneous mutations.

However, current knowledge is not sufficient to explain why only a proportion of individuals exposed to environmental carcinogens or carrying genetic mutations develop carcinomas nor has a consensus been reached regarding these phenomena. Obviously, other factors must be taken into consideration and consequently, the human microbiome is now receiving increased attention by cancer researchers and clinician scientists. To the best of our knowledge, microorganism dysbiosis or invasion may induce or result in chronic inflammatory infection, which has been implicated in the pathogenesis of various human cancers. For instance, *Helicobacter pylori* is associated with gastric adenocarcinoma and mucosa-associated lymphoid tissue (MALT) lymphoma^13^, human papilloma virus is associated with cervical cancer^14^, hepatitis B and hepatitis C viruses are associated with hepatocellular carcinoma^15^ and recently, *Fusobacterium nucleatum* has been associated with colorectal carcinoma^16,17^. Recently, some studies have demonstrated that microbial dysbiosis may be associated with human breast cancer^18,19^. In contrast, the association of microbiota and chronic inflammation with human ovarian cancer initiation and progression has received little attention. A population-based study conducted by Hui-Wen Lin *et al*. suggested an increased risk of ovarian cancer in women with pelvic inflammatory disease^20^. Some groups have also reported that *Chlamydia trachomatis* and *Mycoplasma genitalium* may have an association with epithelial ovarian tumors^21,22^.

Nevertheless, no comprehensive study of microbiota in ovarian cancer has been reported. Given the incomplete understanding of ovarian cancer etiology and the association of chronic infection and inflammation with increased risk of ovarian neoplasm, we hypothesized that microbial composition change may have an association with human ovarian cancer. Here, we performed 16S rRNA sequencing in order to analyze and characterize the overall structure of microbiota in patients with ovarian cancer in comparison to normal distal fallopian tube tissues with the goal of identifying key bacterial phylotypes, potential bacterial biomarkers and possible mechanisms underlying the development of ovarian carcinoma.

## Results

### Decreased bacterial diversity in ovarian cancer tissues

**Figure 1:**
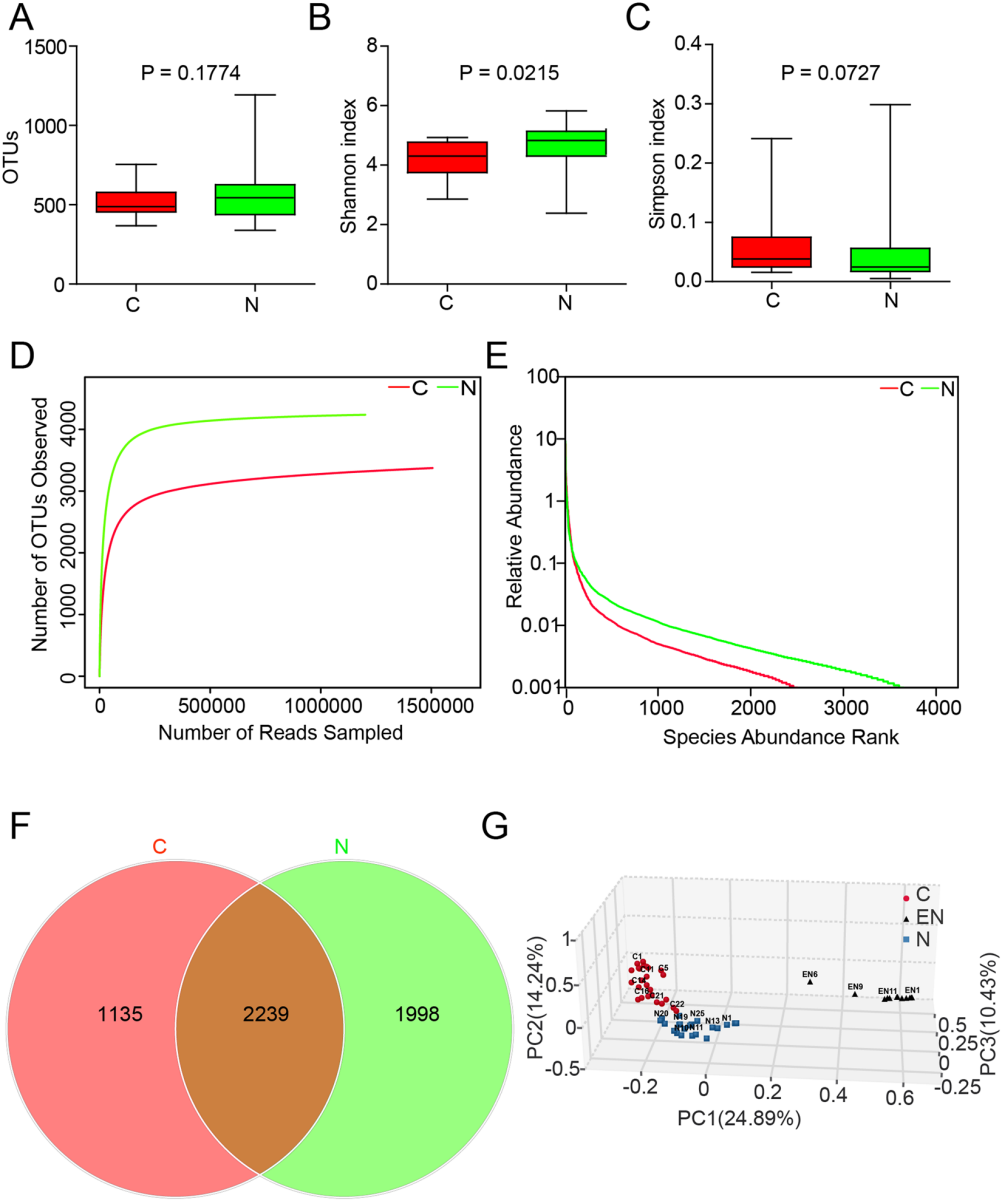
Comparison of the structures of the tissues microbiota of the ovarian cancer (C group) and normal distal fallopian tube. (**A**) Number of OTUs was statistically calculated between C group and N group. There was not statistically different between the two groups. (**B**) Shannon and (**C**) Simpson indices were used to estimate the level of diversity of the microbiota of C group and N group. (**D**) Rarefaction curves were used to estimate the richness of C group and N group at a 97% similarity level. The vertical axis shows the number of OTUs that were expected to be found after sampling the number of tags or sequences shown on the horizontal axis. (**E**) The Rank-Abundance Curve based on the results of OTUs analysis between C group and N group. N group exhibited the relative more abundant species than in C group. The horizontal axis presents the OUT rank, the vertical axis presents the relative abundance of the OUT rank. (**F**) Venn diagrams demonstrating 97% OUT cluster overlap identified in the tissues microbiota of C group and N group. (**G**) The differences in microbial communities among the C group, N group and environmental negative control (EN) as demonstrated by a principal co-ordinates analysis (3D-PCoA) plot based on the unweighted UniFrac metric. Data were shown as the mean and SEM.

Thus far, the ovarian cancer microbiome has not been investigated. In this study, we employed 16S rRNA sequencing to survey the ovarian microbiota in normal distal fallopian tube tissues (N group) and ovarian tumor tissues (C group) from all 50 patients and to make the result more reliable, 12 environmental negative control (EN group) samples were also collected where those fifty patients of both N group and C group received operation from four operating rooms randomly. The patients’ information is shown in Table 1. The sequencing results of these patients produced 3,407,042 raw sequences with a median read length of 438 base pairs, and the average read length ranged from 356.9 to 445.41. After quality trimming and removal of chimeric sequencing, 2,706,457 high-quality reads remained, accounting for 79.4% of the valid reads with an average yield of 54,129 reads (ranging from 6,154 to 96,215) per barcoded sample for downstream analysis. The summary information is shown in Table 2, and the detailed characteristics of each sample are shown in Table S1. From the tables, we found that 7,611 unique sequences were obtained from the two groups, representing all of the phylotypes observed in the ovarian tissue microbiota. The Good’s coverage values approached 99.99% in the two groups, indicating that the sequencing depth was sufficient for the discovery and investigation of the ovarian tissue microbiota. The estimators of community richness (Chao 1 and ACE) and diversity index (Shannon and Simpson) are shown in Table 2. The average number of operational taxonomic units (OTUs) detected did not vary significantly between the two groups (*P* = 0.1774, Fig. 1A). However, the evenness of the communities was significantly different (Adonis testing, R^2^ = 0.15715, *P* < 0.001). There were statistically significant differences between the Shannon indexes of the C and N group, demonstrating slightly decreased diversity in microbiota of the C group relative to the N group (*P* = 0.0215, Fig. 1B), although the Simpson indexes were not significantly different (*P* = 0.0727, Fig. 1C). Rarefaction curve analysis also showed that the species richness of the C group had a tendency to be lower than that of the N group (Fig. 1D). The rank-abundance curves for the bacterial communities revealed that N group had a longer right-hand tail, indicating that greater richness and more uniform distribution in bacterial communities compared to the C group (Fig. 1E). Furthermore, a Venn diagram representing the shared OTUs between the two groups was developed at a 97% similarity level. The results demonstrated that 2,239 of 5,372 OTUs were shared between the two groups, accounting for 41.7% of the total richness, while the number of N group-specific species is 1,998 and the number of C group specific-species is 1,135 (Fig. 1F). Finally, to evaluate the extent of similarity among the N group, C group and environmental negative group (EN group), beta-diversity analysis was performed using the unweighted UniFrac method, and subsequent principal co-ordinate analysis (PCoA) was performed. The results showed that the microbiota of the three groups could be distinctly separated using 3D-PCoA, although with several individual variations (Figs 1G and S1). These results indicate that a decreased bacterial diversity is observable in ovarian cancer tissues compared to normal distal fallopian tube tissues. Moreover, the environmental microbiota composition has little hybird effect on the composition of N group and C group, thus we may carefully draw a conclusion that the pelvic microbiota of ovarian cancer patients is different from that of non-cancer patients.

**Table 1:** Demographic information of subjects in the discovery and validation phases.

Variables	Discovery phase			Validation phase		
	Ovarian cancer (n = 25)	Control group (n = 25)	P value	Ovarian cancer (n = 46)	Control group (n = 41)	P value
Age(mean ± SD)	54.5 ± 7.3	48.2 ± 7.7	0.967	50.5 ± 10.0	47.3 ± 8.2	0.122
**Differentiation**						
Well	0			0		
Moderate	2			6		
Poor	23			40		
**FIGO stage**						
IIIa-IIIc	24			44		
IV	1			2

**Table 2:** Comparison of phylotype coverage and diversity estimation of the 16S rRNA gene libraries at 97% similarity from the 16S rRNA sequencing analysis.

Group	No. of Reads	No. of OTUs^a^	Good’s (%)^b^	Richness estimator				Diversity index		
				ACE	95% CI	Chao1	95% CI	Shannon	Simpson	Evenness^c^
C	1505344	3374	99.98%	3568	3523–3626	3697	3604–3828	5.01	0.0244	0.616617
N	1201113	4237	99.99%	4272	4260–4293	4292	4269–4331	5.97	0.0152	0.715388

^a^The operational taxonomic units (OTUs) were defined at the 97% similarity level.

^b^The coverage percentage (Good’s), Richness estimators (ACE and Chao 1) and Diversity index (Shannon and Simpson) were calculated using Good’s method and the mother program, respectively.

^c^The Shannon index of evenness was calculated with formula E = H/ln(S), where H is the Shannon diversity index and S is the total number of sequences in that group.

### Microbial composition and alterations are associated with ovarian cancer

The taxonomy of the including ovarian cancer and normal distal fallopian tube tissues was assessed by a taxon-dependent analysis using the RDP classifier. In total, 41 phyla were identified in the C group and 45 phyla were found in the N group. Regardless of much lower proportions (<1% of the total taxon abundance), the most frequent taxa identified in the C group were *Proteobacteria*, *Firmicute*, *Bacteroidetes*, *Actinobacteria* and *Bacteria_unclassified*, whereas representative sequences of of *Proteobacteria*, *Firmicute*, *Bacteroidetes*, *Actinobacteria*, *candidate_division_TM7*, *Chloroflexi*, *Acidobacteria*, *Fusobacteria* and *Bacteria_unclassified* were abuntant in the N group. Significant differences were found in the relative proportions of certain phyla: *Proteobacteria*, *SM2F11* and *GOUTA4*, were significantly upregulated in ovarian cancer tissues (*P* < 0.001). While *Firmicute*, *Candidate_division_TM7*, *Acidobacteria*, and *Candidate_division_OD1* were significantly downregulated (*P* < 0.001) (Fig. S2A). The sequences obtained from ovarian tumor tissue and normal distal fallopian tube tissues microbiota could be classified into 1,084 genera, with members of 887 genera found in the C group, and 951 were found in the N group. Considering only the high-abundance represented genera (>1% of the total taxon abundances), members of 21 genera were detected in the C group, whereas 18 genera were observed in the N group. Heat mapping showed correlations between the participants and genera abundance observed in ovarian tissue microbiota (Fig. S2B). The sequences obtained from the ovarian tissue microbiota represent 2,202 species, with 1,652 species found in the C group, and 1,854 in the N group. Irrespective of relative low species abundance, 20 represented species were found in the C group, whereas 17 were detected in the N group. The significantly up- and down-regulated species were analyzed using Wilcoxon sum-rank test and visualized by heat mapping (Fig. S2C). It was clearly evident that the aberrant composition of ovarian tissue microbiota may be associated with ovarian cancer.

**Figure 2:**
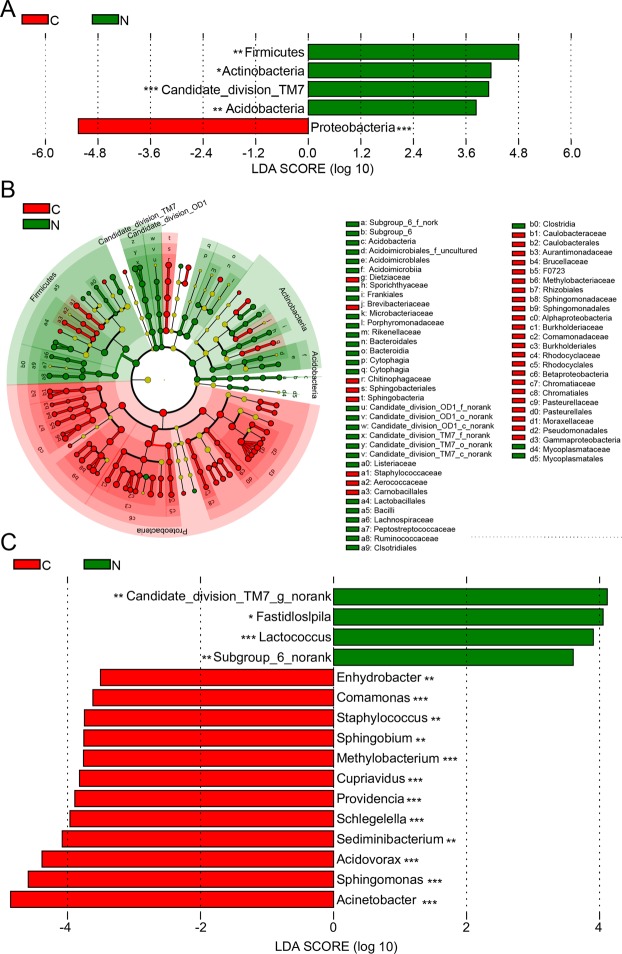
LEfSe was performed to identify the most differentially abundant taxons between ovarian cancer tissues and normal distal fallopian tube tissues. (**B**)Taxonomic representation of statistically and biologically consistent differences between ovarian cancers and normal tissues displayed by a cladogram. (Red) ovarian cancer-enriched taxa; (Green) normal tissues-enriched taxa. The brightness of each dot is proportional to its effect size. Cladogram was calculated by LEfSe, a metagenome analysis approach which performs the liner discriminant analysis following the Wilcoxon sum-rank test to assess effect size of each differentially abundant taxon or OUT. (**A**,**C**) Histogram of the LDA scores for differentially abundant phyla and genera, respectively. Only taxa meeting an LDA significant threshold of 3.5 are shown. *P < 0.05; **P < 0.01; ***P < 0.001.

To identify the specific and key bacterial taxa associated with ovarian cancer, LEfSe was performed to generate the cladogram to identify the predominant bacteria of the microbiota within the two groups, which is shown in Fig. 2B. At the phylum level, *Proteobacteria* and *Firmicutes* were the most dominant taxa in the C and N groups, respectively (Fig. 2A). At the genus level, the proportions of *Acinetobacter*, *Sphingomonas* and *Methylobacterium* were significantly enriched in the C group, whereas *Lactococcus* was dramatically enriched in the N group (Fig. 2C). At the species level, *Acinetobacter_lwoffii* and *Lactococcus_piscium* were significantly enriched in the C and N groups, respectively (Fig. S3). According to LEfSe analysis, these were all key phylotypes differentiating ovarian tumor tissue and normal distal fallopian tube tissue microbiota, and might be potential microbiological markers for discriminating patients with ovarian cancer. Collectively, these differences in microbiota composition change revealed the potential impact on the development of ovarian cancer.

### Identification of key pathogenesis-associated microbes as potential predictors for ovarian cancer

**Figure 3:**
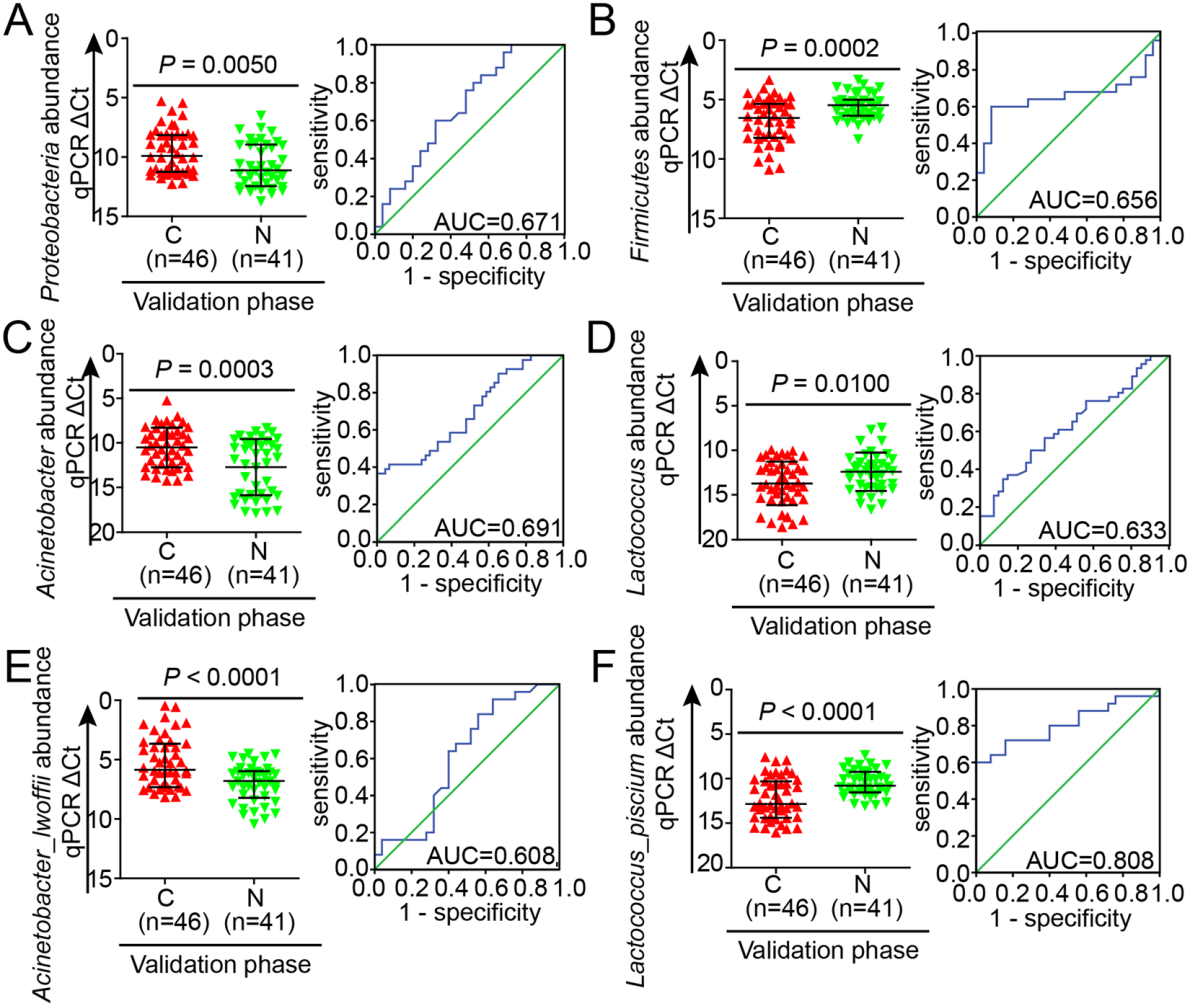
RT-qPCR was used to validate the microbial biomarker candidates in an independent samples and ROC curves constructions. (**A**) *Proteobacteria* was found significantly enriched in ovarian cancer tissues (p < 0.05) and the AUC was 0.671; (**B**) *Firmicutes* was found significantly decreased in ovarian cancer tissues (p < 0.05) and the AUC was 0.656; (**C**) *Acinetobacter* was found significantly increased in ovarian cancer tissues (p < 0.05) and the AUC was 0.691; (**D**) *Lactococcus* was found significantly decreased in ovarian cancer tissues (*P* < 0.05) and the AUC was 0.633; (**E**) *Acinetobacter_lwoffii* was found significantly increased in ovarian cancer tissues (p < 0.05) and the AUC was 0.608; (**F**) *Lactococcus_piscium* was found significantly decreased in ovarian cancer tissues (*P* < 0.05) and the AUC was 0.808.

Using the key phylotypes identified by LEfSe and the significant statistical differences as shown in Fig. 2 and Table S3, to discriminate between the C and N groups. We performed quantitative PCR (qPCR) to validate the potential microbial biomarkers in independently obtained samples, including 46 ovarian tumor tissues and 41 normal distal fallopian tube tissues. At the phylum level, the two microbial biomarkers, *Proteobacteria* and *Firmicutes* showed significant differences between the two groups (*P* < 0.05), in accordance with the results obtained by 16S rRNA sequencing, and yielded ROC-plot AUC values of 0.671 and 0.656, respectively (Fig. 3A,B). At the genus level, *Acinetobacter* was increased (*P* < 0.05), while *Lactococcus* was significantly decreased in ovarian cancer tissues (*P* < 0.05). The corresponding AUC values were 0.691 and 0.633, respectively (Fig. 3C,D). At the species level, the abundance of *Acinetobacter_lwoffii* was markedly enriched in ovarian cancer tissues and *Lactococcus_piscium* was significantly enriched in normal tissues (*P* < 0.001), yielding AUC values of 0.608 and 0.808, respectively (Fig. 3E,F), which were consistent with the results discovered by 16S rRNA sequencing. Our data indicate that excellent diagnostic accuracy was possible only with *Lactococcus_piscium*, which might be linked to other indicators to provide improved early detection of ovarian tumors.

### Functional analysis and inflammation-associated genes profiles in ovarian cancer

**Figure 4:**
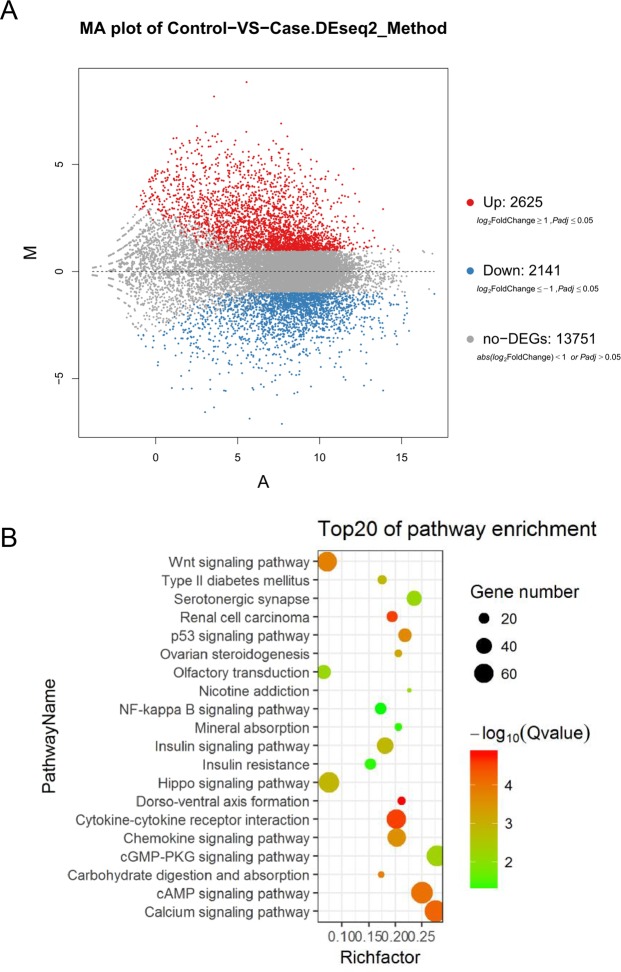
Functional analyses in six ovarian cancer tissues and seven normal distal fallopian tube tissues. (**A**) 2,625 related genes were up-regulated and 2,141 related genes were down-regulated in ovarian cancer tissues compared to normal distal fallopian tube tissues. DEG was shown via MA plot scattergram. (**B**) Enriched KEGG categories in ovarian cancer tissues at a false discovery rate of 5%. X axis represents Rich Factor, Y axis represents Pathway Name, Color represents Q value, the Dot represents the number of DEG (differential gene expression).

The above results demonstrated that the total number and diversity of bacterial communities was dramatically decreased in ovarian cancer tissues compared to that in normal distal fallopian tube tissues. In order to primarily explore the plain mechanism and related signaling pathways, transcriptome sequencing (RNA-seq) analyses were conducted with 6 ovarian cancer tissues and 7 normal distal fallopian tube tissues, and its results suggested a different transcriptional profiles of ovarian cancer tissues compared to normal distal fallopian tube tissues. In general, 2,625 related genes were up-regulated and 2,141 related genes were down-regulated in ovarian cancer tissues (Fig. 4A). We also performed functional inferences from the Kyoto Encyclopedia of Gene and Genomes (KEGG) catalog. As indicated, at an FDR of 0.05, the top 20 signaling pathways were calculated, including P53 signaling pathway, Wnt signaling pathway, Hippo signaling pathway, cAMP signaling pathway, which had been previous found to be associated in the initiation and progression of ovarian cancer. In addition, we also found that inflammation-associated signaling pathways were dramatically activated in ovarian cancer tissues, such as NF-kappa B signaling pathway, Cytokine-cytokine receptor interaction and Chemokine signaling pathway *et al*. (Fig. 4B). Numerous researches have demonstrated that carcinogenesis is inherently a process of inflammation, with many proinflammatory and immunosuppressive pathways acting along the mechanistic details of bacteria-associated carcinogenesis. Thus we hypothesized that the microbial composition change may influence the expression of human antibacterial-response genes in the local tumor microenvironment. Therefore, we performed an inflammation and immune associated array profiles according to a previous study^18^ and compared the human antibacterial-response gene expression profiles between two groups. Among these, the genes involved in the Toll-like receptors (TLR) and NOD-like receptors (NOD) signaling pathways (i.e., TLR4, TLR5, CARD6, NOD1 and NLRP1, *et al*.) were significantly reduced in ovarian cancer tissues, while other genes increased in ovarian cancer tissues (i.e., TLR6, MYD88 and NLRP3, *et al*.) (Fig. S4). These data indicated significant signaling pathways and antibacterial-response genes involved in ovarian cancer initiation and progression. We speculate that the microbial composition change may contribute to impact on the local immune microenvironment of the tumor and thus influence the status of the malignant ovarian tumor. However, the detailed mechanisms involved in the initiation or progression of ovarian cancer require further research.

## Discussion

Epithelial ovarian cancer (EOC) is a highly heterogeneous and multifactorial diseases, whose aetiology and precursor lesions are still poorly understood^23^. Over the last decades, microorganisms inhibiting in our bodies have been increasingly investigated as possible tumor initiators or promoters^24^. Herein, we used next-generation sequencing techniques to provide a comprehensive survey of the microbiota in ovarian tumor tissues and normal distal fallopian tube tissues. In addition, we explored the possible gene expression profiles and associated signaling pathways via transcriptome sequencing. The Good’s coverage approached 99.9%, indicating that the 16S rRNA sequencing identified in the two groups represented the majority of the bacteria in the ovarian tissue microbiota. Meanwhile, an average of 54,129 reads per sample passed all quality filters, accounting for 79.4% of the raw sequence reads. A large number of taxa were identified at relatively low abundance, which may influence the overall bacterial diversity and richness of the ovarian tissue microbiota. The Shannon index were significantly different (*P* = 0.0215), although there was no significant difference in the number of OTUs detected in the two groups (*P* > 0.05). However, the evenness of the two communities was markedly different (Adonis testing, R^2^ = 0.15715, *P* < 0.001). These data, combined with the results of PCoA analysis, indicate that there is a reduction in the composition of the bacterial community (diversity and richness) in ovarian cancer tissues compared to that in normal distal fallopian tube tissues.

Through LEfSe analysis, *Proteobacteria* was found to be the most abundant phylum in ovarian cancer tissues, which was distinct from normal distal fallopian tube tissues in our study, and from the microbiota of the vagina^25^, oral cavity^26^, skin^27^, bladder^28^, and gastrointestinal tract^29^. Interestingly, our finding was similar to observations in breast cancer tissues by Xuan *et al*.^18^. This suggests that the highly heterogeneity exhibited by ovarian cancer potentially correlates with breast cancer. However, the human microbiota composition and diversity may vary over time and by anatomical position^30^. Previous studies have illustrated that *Proteobacteria*, with an outer membrane composed of lipopolysaccharide, had the potential for direct interaction with intestinal mucosal cells via bacterial secretion systems such as T2SS or T3SS^31,32^. An increase in animal microbiota communities had also been reported in experimentally induced colitis and in patients suffering from irritable bowel disease^33^. While another report demonstrated that *Proteobacteria* may participate in the metabolic by-products of fatty acid metabolism, in combination with host genes involved in fatty acid biosynthesis^34^. However, *Firmicutes*, which has been demonstrated to enhance energy yields from the diet, was significantly enriched in intestinal lumen^35–37^. In addition, research has suggested that some genera within *Firmicutes* play a vital role in the production and metabolism of butyrate, which may protect against early tumorigenic events in intestinal mucosa^38–40^. These data demonstrated that dysbiosis of *Proteobacteria* and *Firmicutes* ratios may influence ovarian tumorigenesis if they gave rise to persisted infection. *Helicobacter pylori* is firmly established as a “cancer microbe”, promoting gastric cancer and MALT lymphoma^13,41^. *Fusobacterium nucleatum* infection is prevalent in colorectal carcinoma and potentiates intestinal tumorigenesis^16,17,42^. Shanmughapriya *et al*. suggested that *Chlamydia trachomatis* infection was associated with the initiation or progression of ovarian cancer^22,43^. However, the interactions between bacteria and their hosts are incredibly complex. Not all bacteria are pathogenic or can promote cancer progression as a whole or as a directly pro-oncogenic microbe; some bacteria have probiotic effects to maintain the status of our health. At the species level, *Acinetobacter_lwoffii* species belonged to *Acinetobacters* genera was found significantly increased in ovarian cancer tissues. This species is considered to be normal flora that inhabits the oropharynx, human skin and the perineum, and has tropism for urinary tract mucosa^44^. However, bacteremia may occur in immunocompromised patients, especially tumor patients^45^. It has been suggested that some proteins secreted by *Acinetobacter_lwoffii* may be candidate biomarkers for early diagnosis of oral squamous cell carcinoma (OSCC)^46^. In contrast, *Lactococcus_piscium* species belonged to *Lactococcus* genera, which was generally regarded as gut commensal with probiotic features, a bioprotective bacterial, was significantly decreased in ovarian cancer tissues. *Listeria monocytogenes* can be inhibited by *L. piscium* via a cell-to-cell contact-dependent manner^47^. *L. piscium* may be a microbial biomarker for the discrimination of malignant ovarian tumor versus benign tissue as its AUC approached 0.808. However, the microbial composition of tumors is very complex, and its use as a diagnostic tool requires further research.

Microbiota can profoundly influence many aspects of host physiology, such as activating the immune system, regulating metabolism and promoting cancer progression. The interplays between microbiota and the initiation and progression of cancer are numerous and complex, as seen with the Toll-like and Nod-like receptor signaling pathways^48,49^. Mechanistically, many signaling pathways including P53 signaling pathway, Wnt signaling pathway, Hippo signaling pathway, cAMP signaling pathway and even inflammation-associated signaling pathways were significantly regulated in ovarian cancer tissues compared to normal tissues. However, the detailed mechanism why the majority of the antibacterial-response genes were upregulated or downregulated in ovarian cancer tissues was still unclear. We speculated that some kinds of bacterial may use a wealth of mechanisms to evade or overcome the host immune system in order to survive inside of the host and maintain a persistent infection. A study from Bei Li *et al*. supposed that *Yersinia pestis* may defense against host immunity and could inhibit the production of proinflammatory cytokines and attenuate the host’s adaptive immunity to promote dissemination and persisted infection^50,51^. Taken together, our data suggest that microbial dysbiosis may have a potential role in manipulating the initiation or progression of ovarian cancer through suppression of host inflammatory and immune responses, resulting in immunosuppression in the local microenvironment of the tumor. In turn, the tumor microenvironment may create a milieu favorable to the persistence for specific bacteria, ultimately resulting in a permissive signal for ovarian tumorigenesis.

Several limitations of our present study should be acknowledged. First, the distal fallopian tube tissues used in this study were obtained from patients of adenomyoma or myoma of the uterus undergoing uterine plus bilateral salpingo-oophorectomy and thus cannot be considered completed normal. However, this did not affect the comparison between the two groups. Second, whether this variation in bacterial abundance is a causative factor for carcinogenesis or a consequential reflection of the tumor microenvironment needs to be further explored. Third, longitudinal studies should also be conducted to determine whether the key phenotypes are true microbial biomarkers.

In summary, our study, for the first time, compared the composition and diversity of bacterial communities in ovarian cancer tissues based on 16S rRNA sequencing. At the same time, our study also challenges the dogma that the upper reproductive tract is normally a sterile area, at least in ovarian cancer patients. Interestingly and accordingly, our finding is similar to what was recently observed by Sagarika Banerjee *et al*.^52^ and another paper reported by Shana M. Mile *et al*.^53^ They both suggest that the upper reproductive tract has unique microbiome and is not sterile. Furthermore, the consequences of transcriptome sequencing analyses and antibacterial-response gene expression profiles explored the potential mechanisms of pathogenesis. Based on our results, further investigations into the role of microbes in ovarian cancer are highly recommended.

## Methods

### Subjects’ enrollment, samples collection and experimental designation

The protocols used for the present study were approved by the Ethics Committee of Tongji Hospital, Tongji Medical College, Huangzhong University of Science and Technology. The methods were carried out in accordance with the approved guidelines and written informed consents were obtained from all participants prior to enrollment. Fresh ovarian cancer tissues were recruited from 71 women who were diagnosed with high-grade serous ovarian cancer via quickly frozen biopsy during operation. 66 fresh normal fallopian tube fimbria tissues were obtained from patients of adenomyoma or myoma of uterus undergoing uterine plus bilateral salpingo-oophorectomy. Of these, 25 ovarian cancer tissues (C group) and 25 normal distal fallopian tube tissues (N group) were selected for discovery of this study via 16S rRNA sequencing, whereas the remaining 46 ovarian cancer tissues and 41 normal tissues were collected for validation through quantitative PCR (qPCR). All the pathological type of the tissues was validated by two independent clinical pathologists in a blind fashion. The normal tissues were also verified to be tumor-free. After excision, the fresh tissues were immediately placed in a sterile vial on ice and homogenized within 30 minutes. As an environmental negative control (EN group), when an operation began we opened a sterile vial and kept the vial open until the tissue was collected, and the samples of EN group were treated the same as that of N group and C group such as homogenization, DNA isolation and all process of 16S sequencing. In total, 12 EN samples were collected and sequenced. Patients with a history of the use of antibiotics, probiotics, prebiotics, or synbiotics in the previous at least three months or active bacteria, fungal, viral or sexually transmitted infections in other parts of the body were excluded from this study. Due to advanced stage diagnosis of the majority of ovarian cancer, staged IIIC or IV (International Federation of Gynecology and Obstetrics staging) and conforming to HGSOC histopathological type were gathered.

### Extraction, amplification of bacterial genomic DNA and 16S rRNA sequencing

Total genomic DNA was extracted from tissues using QIAamp DNA Mini Kit (QIAGEN, Hilden, Germany) according to the manufacturer’s instructions with slight modifications as described previously^18,19^. All operations were performed in a UV-irradiated clean room. DNA was stored at −20 °C until further analysis. The bacterial genomic DNA was amplified with the 338 F (5′-ACTCCTACGGGAGGCAGCA-3′) and 806 R (5′-GGACTACHVGGGTWTCTAAT-3′) primers specific for the V3-V4 hypervariable regions of the 16S rRNA genes^54^. To sequence our amplicon, dual indices and Illumina sequencing adapters were attached using the Nextera XT Index Kit and purified the amplicon using AMPure XP beads^55^. PCR was performed with 10 ng template, 0.3 μlExTaq Polymerase (TaKaRa Bio, Japan), 2 μl 10 × Extaq Buffer, 0.8 μl Forward Frimer (5 μM), 0.8 μl Reverse Primer (5 μM) on an ABI GeneAmp 9700 cycler. The cycling parameters were as follows: 5 min of denaturation at 95 °C followed by 30 cycles of 30 seconds at 95 °C (denaturation), 30 seconds for annealing at 52 °C and 45 seconds at 72 °C for elongation, with a final extension at 72 °C for 10 min. Triplicate PCR reactions were performed on each sample. Amplified products from stool samples were verified by gel electrophoresis using 5 ml of the PCR reaction mixture in a 2.0% agarose gel. The

PCR products were purified by using the AxyPrepDNA Gel extraction kit (Axygen, US) and quantified on QuantiFluor TM-ST Fluorometer (Promega, US). The products from different samples were mixed at equal ratios for sequencing using the Illumina MiSeq system (Illumina MiSeq, USA), according to the manufacturer’s instructions.

### RNA sequencing data analysis

Six ovarian serous cancer tissues staged IIIC or IV and seven normal distal fallopian tube tissues from patients of myoma of uterus undergoing uterine plus bilateral salpingo-oophorectomy. Total RNA was extracted from seven fresh-frozen normal fallopian distal tube and six ovarian cancers tissues selected for gene expression analysis, respectively. And then total RNA was converted to cDNA using iScript cDNA synthesis kit (Bio-Rad). RNA sequencing platform was BGISEQ-500 and data analysis was conducted using standard method in BGI-Shenzhen (Shenzhen 518083, China).

### Bioinformatics and statistical analysis

Raw sequencing reads obtained from the sequencer were denoised using the Pre.cluster tool and detailed sequence processing was described as previously^56^. The high-quality reads were clustered into operational taxonomic units (OTUs) using Mothur^57^. The OTUs that reached a 97-nucleotide similarity level were used for alpha diversity (Shannon, Simpson index) and richness analysis (ACE and Chao1), Good’s coverage, Venn diagram, rarefaction curve analysis and Principal coordinate analysis using Mothur software package. The heat mapping was constructed on the basis of the relative abundance of OTUs using R (version 2.15; The R Project for Statistical Computing; http://www.R-project.org). Taxonomy-based analyses were performed by classifying each sequence using Ribosomal Database Project (RDP) database (http://rdp.cme.msu.edu/) with a 50% bootstrap score^58^. A metagenomic biomarker discovery approach was employed with LEfSe [linear discriminant analysis (LDA) coupled with effect size measurement] which performed a nonparametric Wilcoxon sum-rank test followed by LDA analysis using online software (http://huttenhower.sph.harvard.edu/galaxy/) to assess the effect size of each differentially abundant taxon^59^. The t-test and Wilcoxon sum-rank test were performed using SPSS (version 21.0; SPSS Inc., Chicago, IL, USA) for statistical analysis. The Wilcoxon test was also used to compare the microbiological markers between the two groups. Meanwhile, for each biomarker, we constructed the received operating characteristic curve (ROC) and computed the area under the curve (AUC) value. *P* < 0.05 was considered statistically significant.

## Supplementary information

Supplementary information
