# Supplementary material for: The biodiversity Composition of Microbiome in Ovarian Carcinoma Patients
Source: Sci Rep. 2019 Feb 8;9:1691. doi: 10.1038/s41598-018-38031-2 (PMC6368644; doi:10.1038/s41598-018-38031-2)
Supplement: Supplementary file 1 — Supplementary information [file 41598_2018_38031_MOESM1_ESM.pdf]

Supplementary Information

The biodiversity Composition of Microbiome in Ovarian Carcinoma Patients

Bo Zhou[1,*], Chaoyang Sun[1,*], Jia Huang[1], Meng Xia[1], Ensong Guo[1], Na Li[1], Hao Lu[1], Wanying Shan[1], Yifan Wu[1], Yuan Li[1], Xiao Xu[1], Danhui Weng[1], Li Meng[1], Junbo Hu[1], Qinglei Gao[1], Ding Ma[1] & Gang Chen[1]

[1]Cancer Biology Research Center, Tongji Hospital, Tongji Medical College, Huazhong University of Science and Technology, Wuhan, Hubei 430030, China

*These authors contributed equally to this work.

Running title: Microbial Dysbiosis in Human Ovarian Cancer

Correspondence to: Dr. Gang Chen, Cancer Biology Research Center, Tongji Hospital, Tongji Medical College, Huazhong University of Science and Technology, Wuhan, Hubei 430030, China; E-mail: chengang@tjh.tjmu.edu.cn

Dr. Ding Ma, Cancer Biology Research Center, Tongji Hospital, Tongji Medical College, Huazhong University of Science and Technology, Wuhan, Hubei 430030, China; E-mail: dma@tjh.tjmu.edu.cn

Figure S1 Microbiota composition in 25 ovarian carcinoma patients (C group), 25 normal distal fallopian tube tissues (N group) and environmental negative control samples (EN group), identified by 16S rRNA sequencing. The relative abundances of bacterial phyla identified in each tissue samples and EN were visualized by bar plots. Each bar represents a subject and each colored box a bacterial taxon. The height of a colored box represents the relative abundance of that organism within the sample. Taxa present in less than 1% abundance in a given sample are displayed in the "others" at the top of the graph.

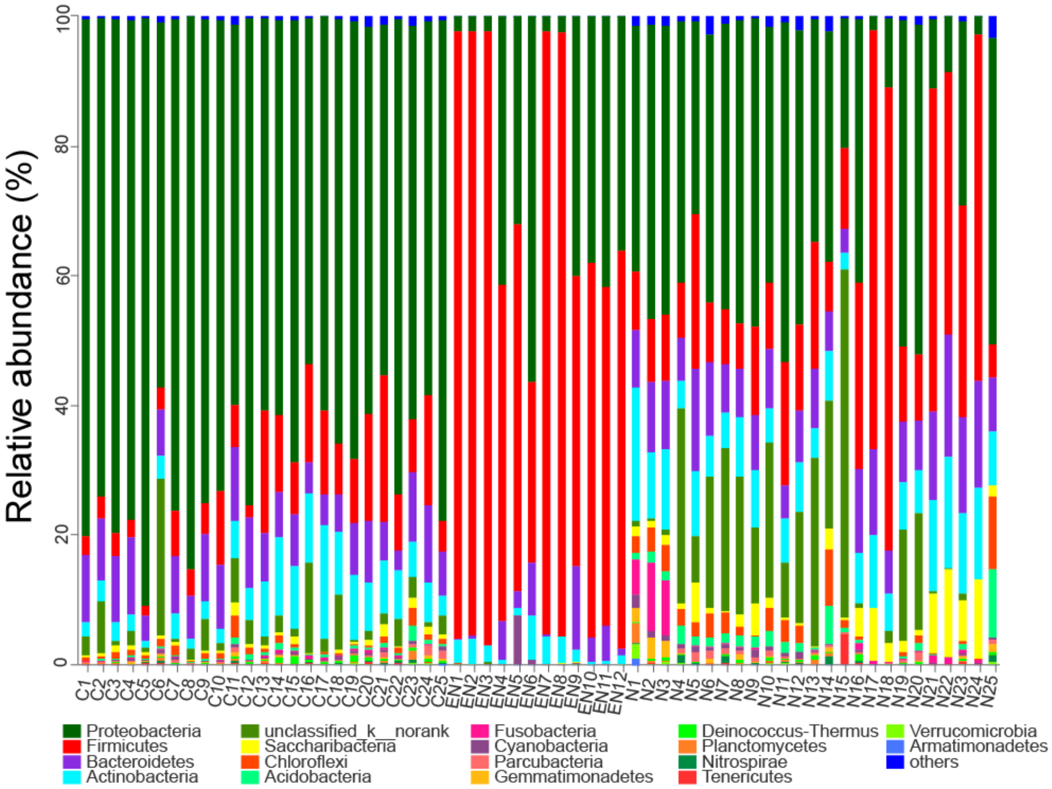

Figure S2 Heatmap of percentage of the significantly statistically different phyla (a), genera (b) and species (c) in the tissues of each sample based on 16S rRNA sequencing.

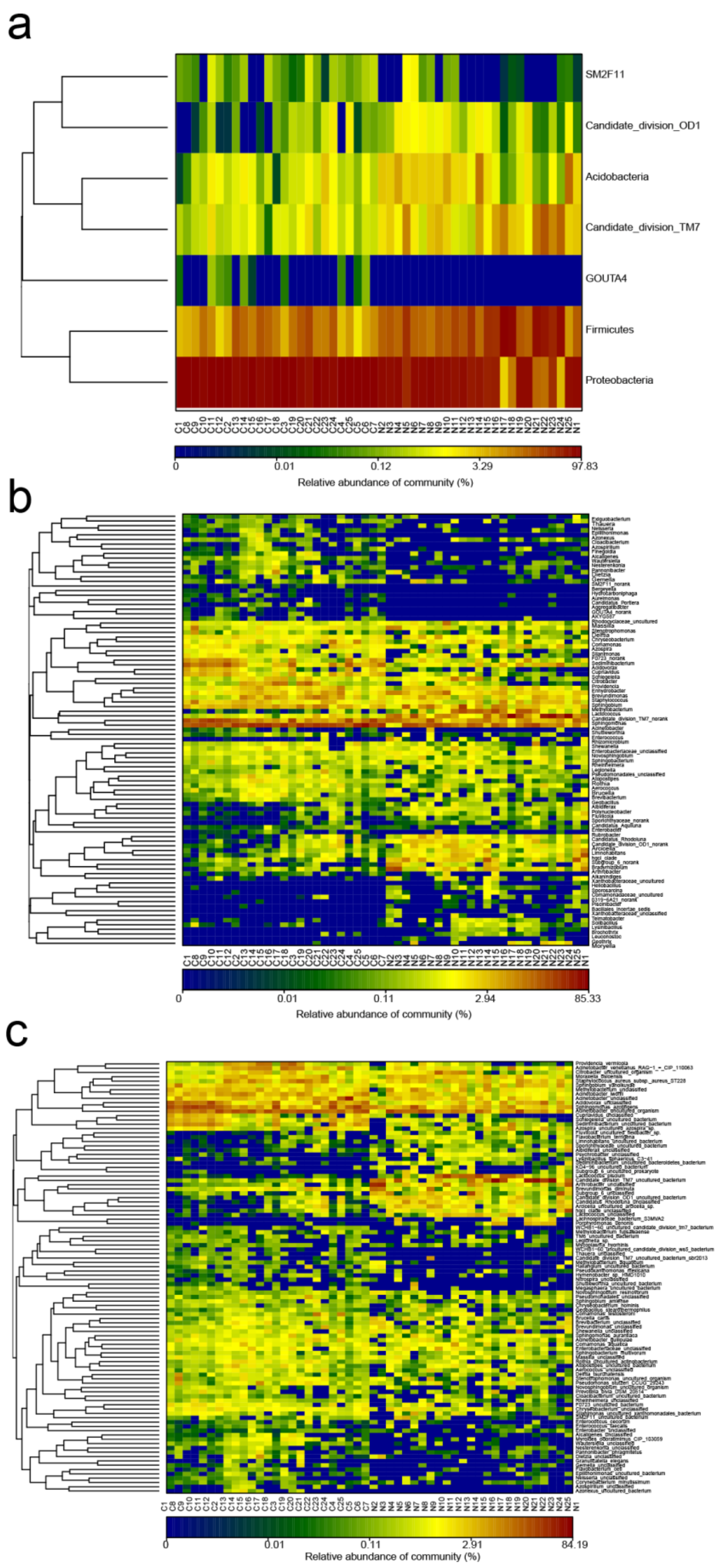

Figure S3 Histogram of the LDA scores for differentially abundant species. Only taxa meeting a LDA significant threshold of 3.5 are shown.

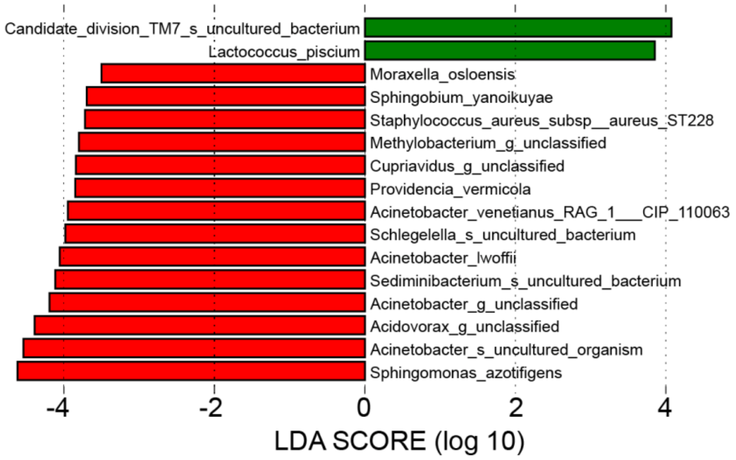

Figure S4 Heatmap of inflammation and immune associated gene profiles using non-supervised hierarchical clustering.(con. represents normal distal fallopian tube tissues. Case represents ovarian cancer tissues.)

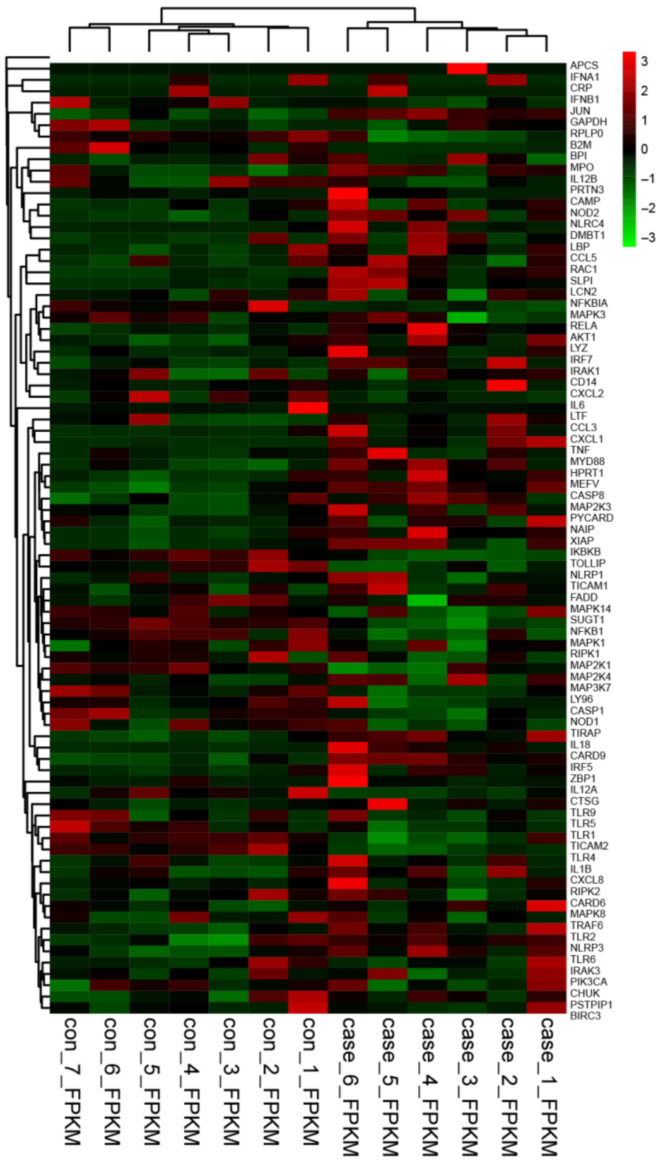

Table S1 Comparison of the phylotype Good' s coverage, the richness estimators and the diversity index of the 16S rRNA gene libraries of individuals at a 97% similarity level with Mothur analysis

Sample ID	No. of Reads	No. of OTUs	Ace (lci, hci)	Chao 1 (lci, hci)	Good's Coverage	Shannon (lci, hci)	Simpson (lci, hci)
C1	38664	491	523 (510,545)	533 (513,570)	0.998345	3.64 (3.62,3.65)	0.0756 (0.0741,0.077)
C2	62292	422	465 (448,492)	468 (446,509)	0.999005	3.8 (3.79,3.81)	0.0426 (0.0421,0.0432)
C3	51819	523	562 (547,589)	610 (569,688)	0.998746	3.74 (3.72,3.76)	0.0704 (0.0692,0.0716)
C4	29050	481	549 (525,587)	568 (531,633)	0.997108	4.03 (4.01,4.06)	0.0645 (0.0626,0.0664)
C5	31372	611	679 (655,715)	707 (666,777)	0.997227	4.69 (4.67,4.71)	0.0354 (0.0343,0.0364)
C6	79551	690	770 (743,809)	813 (763,895)	0.998718	3.76 (3.75,3.78)	0.0797 (0.0786,0.0807)
C7	75396	538	597 (574,633)	664 (606,773)	0.999005	3.88 (3.87,3.89)	0.0603 (0.0594,0.0612)
C8	76552	594	622 (610,645)	634 (613,677)	0.999386	4.76 (4.74,4.77)	0.021 (0.0207,0.0213)
C9	81570	630	666 (651,693)	679 (655,728)	0.999338	4.85 (4.84,4.86)	0.0254 (0.0249,0.0259)

C10	81053	659	692<br>(678,716)	710<br>(684,761)	0.999358	4.89<br>(4.88,4.91)	0.0204<br>(0.0201,0.0207)
C11	87961	461	599<br>(559,655)	578<br>(522,685)	0.999216	4.3<br>(4.29,4.31)	0.0372<br>(0.0366,0.0378)
C12	90756	368	385<br>(376,406)	383<br>(373,409)	0.999747	4.38<br>(4.37,4.39)	0.0243<br>(0.024,0.0246)
C13	28949	460	499<br>(482,527)	537<br>(499,614)	0.998066	4.69<br>(4.67,4.71)	0.0264<br>(0.0256,0.0272)
C14	77061	479	508<br>(495,531)	524<br>(501,573)	0.999390	3.58<br>(3.57,3.59)	0.0865<br>(0.0853,0.0877)
C15	92987	754	1210<br>(1127,1312)	1058<br>(952,1222)	0.998215	4.78<br>(4.76,4.79)	0.0252<br>(0.0249,0.0256)
C16	40172	563	817<br>(760,891)	781<br>(692,931)	0.997187	4.8<br>(4.78,4.82)	0.0222<br>(0.0216,0.0227)
C17	59784	485	568<br>(538,614)	566<br>(531,629)	0.998679	4.58<br>(4.57,4.6)	0.0246<br>(0.0242,0.025)
C18	82628	434	466<br>(451,494)	485<br>(457,545)	0.999492	3.14<br>(3.12,3.16)	0.2414<br>(0.2382,0.2446)
C19	46215	391	413<br>(402,435)	421<br>(404,460)	0.999243	4.78<br>(4.77,4.79)	0.0166<br>(0.0162,0.0169)
C20	31948	415	448<br>(432,478)	444<br>(428,480)	0.998842	4.93<br>(4.92,4.95)	0.0156<br>(0.0152,0.016)

C21	61693	528	580<br>(560,611)	601<br>(568,661)	0.998833	3.59<br>(3.57,3.61)	0.0842<br>(0.0829,0.0854)
C22	43586	488	523<br>(508,550)	543<br>(515,601)	0.998830	4.46<br>(4.44,4.48)	0.0383<br>(0.0374,0.0392)
C23	39867	476	539<br>(518,572)	550<br>(520,601)	0.997667	2.86<br>(2.84,2.89)	0.1505<br>(0.1481,0.1528)
C24	72473	532	579<br>(560,611)	600<br>(567,665)	0.999186	4<br>(3.98,4.01)	0.0742<br>(0.0731,0.0754)
C25	41945	449	597<br>(557,651)	581<br>(523,685)	0.997974	4.12<br>(4.11,4.14)	0.0492<br>(0.0481,0.0502)
N1	34869	1170	1268<br>(1242,1303)	1305<br>(1262,1368)	0.994953	5.27<br>(5.24,5.29)	0.0246<br>(0.0238,0.0254)
N2	96215	1087	1131<br>(1113,1160)	1139<br>(1114,1187)	0.999376	5.54<br>(5.52,5.55)	0.0186<br>(0.0182,0.019)
N3	84588	593	641<br>(622,673)	660<br>(628,721)	0.999255	4.83<br>(4.82,4.84)	0.0317<br>(0.031,0.0324)
N4	91573	496	549<br>(528,586)	580<br>(539,661)	0.999345	4.62<br>(4.61,4.63)	0.025<br>(0.0247,0.0254)
N5	55318	544	617<br>(590,659)	619<br>(586,679)	0.998662	5.01<br>(5,5.02)	0.0179<br>(0.0175,0.0184)
N6	72186	533	564<br>(550,590)	571<br>(551,613)	0.999377	4.89<br>(4.88,4.9)	0.0209<br>(0.0205,0.0213)

N7	21153	427	485<br>(463,520)	488<br>(460,537)	0.996644	4.64<br>(4.62,4.66)	0.0213<br>(0.0207,0.0219)
N8	24092	449	531<br>(502,575)	532<br>(497,592)	0.996389	4.83<br>(4.81,4.85)	0.016<br>(0.0156,0.0165)
N9	32876	614	696<br>(668,738)	716<br>(674,787)	0.997141	4.97<br>(4.95,4.99)	0.0278<br>(0.0268,0.0288)
N10	29852	457	506<br>(487,540)	572<br>(515,684)	0.997890	4.64<br>(4.62,4.66)	0.0284<br>(0.0276,0.0292)
N11	20122	586	653<br>(630,688)	677<br>(639,741)	0.995478	5.26<br>(5.24,5.28)	0.0113<br>(0.0109,0.0116)
N12	6154	449	544<br>(514,587)	539<br>(505,594)	0.981963	4.94<br>(4.91,4.98)	0.0161<br>(0.0152,0.0171)
N13	32766	944	1000<br>(980,1030)	1031<br>(995,1093)	0.997284	5.79<br>(5.78,5.81)	0.0121<br>(0.0116,0.0126)
N14	36951	545	574<br>(562,596)	581<br>(563,617)	0.998566	4.36<br>(4.34,4.38)	0.0585<br>(0.0568,0.0602)
N15	34025	510	541<br>(528,564)	558<br>(534,605)	0.998442	4.81<br>(4.8,4.83)	0.0219<br>(0.0212,0.0225)
N16	82331	382	493<br>(457,546)	552<br>(484,664)	0.998652	2.38<br>(2.37,2.4)	0.2276<br>(0.2248,0.2303)
N17	35042	411	488<br>(461,530)	486<br>(454,540)	0.997546	2.67<br>(2.64,2.69)	0.2985<br>(0.2931,0.304)

N18	37889	545	582<br>(567,607)	594<br>(571,639)	0.998390	4.25<br>(4.23,4.27)	0.0535<br>(0.0522,0.0548)
N19	34930	549	595<br>(577,626)	600<br>(576,646)	0.998254	5<br>(4.98,5.02)	0.0177<br>(0.0172,0.0182)
N20	26113	354	384<br>(371,406)	393<br>(373,432)	0.998047	3.3<br>(3.27,3.32)	0.1102<br>(0.1074,0.113)
N21	46424	403	453<br>(434,482)	462<br>(436,509)	0.998384	3.24<br>(3.22,3.26)	0.0764<br>(0.0755,0.0774)
N22	77183	639	688<br>(669,718)	700<br>(672,753)	0.999132	4.5<br>(4.49,4.52)	0.0375<br>(0.0368,0.0382)
N23	40914	339	422<br>(394,465)	443<br>(400,515)	0.997678	2.73<br>(2.71,2.74)	0.1339<br>(0.1316,0.1362)
N24	69870	650	693<br>(674,727)	697<br>(672,750)	0.999356	5.82<br>(5.82,5.83)	0.0051<br>(0.005,0.0052)
N25	77677	1193	1252<br>(1232,1284)	1275<br>(1241,1332)	0.998841	5.67<br>(5.66,5.69)	0.0125<br>(0.0122,0.0128)

Sample ID: N represents that the control group; C represents that the serious ovarian cancer group. Reads: total trimed sequences that divided into the overall OTUs. The number of OTUs, richness estimator (Chao1, Ace), diversity index (Shannon, Simpson), and coverage index (Good’s coverage) are calculated at a 97% similarity level. OTUs: operational taxonomic units; lci: lower confidence interval, hci: higher confidence interval.

Table S2 Primer Sequences Used for Tissues Microbiota Profiling by qPCR*.

Bacterial strains		Primer sequence (5’ to3’)
Phylum *Proteobacteria*	Forward	CCGTGAAGTCGGAATCGCTA
	Reverse	ATCACACCGTGGTAACCGTC
Phylum *Firmicutes*	Forward	CTGATGGAGCAACGCCGCGT
	Reverse	ACACYTAGYACTCATCGTTT
Genus *Acinetobacter*	Forward	GGCGACGATCTGTAGCGGGTCTG
	Reverse	GGTTACCTTGTTACGACTT
Genus *Lactococcus*	Forward	TGTAGGGAGCTATAAGTTCTCTGTA
	Reverse	GGCAACCTACTTYGGGTACTCCC
Species *Acinetobacter_lwoffii*	Forward	GGCGACGATCTGTAGCGGGTCTG
	Reverse	CGAGCAGACAGAGCAGAAGA
Species *Lactococcus_piscium*	Forward	GCTATCCAGCCCTAAGTGA
	Reverse	AAAGGTTAGCTCACCGGCTTTGGGTA
16S rRNA	Forward	GCGTAGGCGGATTATCAAGT
	Reverse	CGGATTTTACCCCTACACCA

*Primers were designed based on “Probe Design Tool” of the ARB Software package (http://www.arb-home.de/). Ludwig W, Strunk O, Westram R, Richter L, Meier H, Yadhukumar, et al. ARB: a software environment for sequence data. Nucleic acids research. 2004;32:1363-71.

Table S3 Phylotypes significantly different between microbiota of ovarian cancer tissues and normal distal fallopian tube tissues.

Taxonomic Rank	Taxon	mean(C)	sd(C)	mean(N)	sd(N)	p-value	q-value
Phylum	Proteobacteria	0.682696	0.100797	0.321644	0.150875	1.42E-09[$]	3.11E-08
Genus	Acinetobacter	0.173228	0.10951	0.030215	0.02409	3.67E-09[$]	2.10E-06
Species	Acinetobacter_uncultured_organism	0.083345	0.034567	0.018387	0.017883	1.17E-08	1.06E-05
Species	Acinetobacter_lwoffii	0.024399	0.015352	0.003184	0.00285	1.84E-08[$]	1.06E-05
Species	Acinetobacter_unclassified	0.037841	0.097702	0.004212	0.003451	4.98E-08	1.39E-05
Species	Acinetobacter_venetianus_RAG-1_=_CIP_110063	0.021904	0.019858	0.002987	0.004587	7.51E-07	7.85E-05
Genus	Sphingomonas	0.092867	0.077248	0.014701	0.011951	1.81E-06	0.000208
Species	Sphingomonas_azotifigens	0.08644	0.077669	0.008084	0.009162	4.54E-07	5.22E-05
Genus	Schlegelella	0.018501	0.016323	0.000777	0.000999	6.03E-08	1.15E-05
Species	Schlegelella_uncultured_bacterium	0.018501	0.016323	0.000777	0.000999	6.03E-08	1.39E-05
Genus	Providencia	0.016298	0.024329	0.000829	0.001341	4.98E-06	0.000316
Species	Providencia_vermicola	0.016298	0.024329	0.000829	0.001341	4.98E-06	0.000301
Genus	Cupriavidus	0.01293	0.014496	0.000229	0.000527	6.07E-06	0.000341
Species	Cupriavidus_unclassified	0.012889	0.014524	0.000196	0.000471	2.37E-06	0.000196
Genus	Methylobacterium	0.025698	0.013783	0.013429	0.023865	5.01E-05	0.001685
Species	Methylobacterium_unclassified	0.016868	0.010942	0.004467	0.005525	4.67E-06	0.000298
Genus	Sphingobium	0.01595	0.015961	0.004849	0.006599	1.80E-05	0.000687
Species	Sphingobium_yanoikuyae	0.013224	0.015643	0.004005	0.005684	1.65E-05	0.000826

Genus	Comamonas	0.010478	0.010943	0.002556	0.003536	4.67E-06	0.000316
Species	Comamonas_aquatica	0.004772	0.003814	0.001589	0.002663	2.77E-05	0.001147
Genus	Enhydrobacter	0.011531	0.008989	0.004617	0.004028	0.000384	0.007325
Species	Moraxella_osloensis	0.011531	0.008989	0.004617	0.004028	0.000384	0.007724
Phylum	Candidate_division_TM7	0.005517	0.004677	0.032836	0.037847	0.000104	0.001146
Genus	Candidate_division_TM7_norank	0.005517	0.004677	0.032836	0.037847	0.000104	0.003137
Species	Candidate_division_TM7_uncultured_bacterium	0.00205	0.003253	0.027	0.039931	0.00037	0.007601
Phylum	Firmicutes	0.084354	0.056886	0.218328	0.192702	0.001041[#]	0.00405
Genus	Lactococcus	0.000466	0.000781	0.016484	0.022114	6.56E-06[$]	0.000341
Species	Lactococcus_piscium	6.93E-05	0.000154	0.013657	0.019263	1.73E-07[$]	2.84E-05
Genus	Fastidiosipila	0.000703	0.001134	0.024966	0.038836	0.04378	0.138354
Phylum	Acidobacteria	0.005164	0.005098	0.016808	0.02218	0.002991	0.009401
Genus	Subgroup_6_norank	0.001425	0.001576	0.008113	0.01324	0.001897	0.019815

Statistical analysis was performed by Wilcoxon rank-sum test. $^{\#}P < 0.01$, $^{\$}P < 0.001$ acted as the key phylotypes that were selected as microbiological markers based on LEfSe and *P* value at different phylogenetic levels to discriminate between C group and N group.

Relative abundances were shown as mean and sd. Data had no statistically significant difference were not shown.
